# Antibiograms of intensive care units at an Egyptian tertiary care hospital

**DOI:** 10.1186/s43168-021-00059-w

**Published:** 2021-03-08

**Authors:** Essamedin M. Negm, Sherif M. S. Mowafy, Ahmad A. Mohammed, Marwa G. Amer, Ahmed E. Tawfik, Ashraf E. S. Ibrahim, Tarek H. Hassan

**Affiliations:** 1 Anesthesia and Surgical Intensive Care Department, Faculty of Medicine, Zagazig University, Zagazig, Egypt; 2 Clinical Pathology Department, Faculty of Medicine, Zagazig University, Zagazig, Egypt; 3 Chest Department, Faculty of Medicine, Zagazig University, Zagazig, Egypt; 4 Zagazig University Hospitals, Zagazig, Egypt

**Keywords:** Intensive care unit, Microorganisms, Antimicrobial resistance, Antibiogram

## Abstract

**Background:**

Intensive care unit (ICU) infection management is a growing challenge, and physicians should have regularly updated antibiograms. The aim of this study was to find out the prevalence of pathogens and to determine their antibiotic susceptibility in different ICUs of an Egyptian tertiary care hospital. This retrospective record-based cross-sectional study was conducted from the first of January to the last of December 2019 with a total of 45,221 diagnostic first-isolate culture/patient obtained from different ICUs in Zagazig University Hospitals. The antibiogram construction was done according to Clinical and Laboratory Standards Institute instructions and a Web-based antibiogram at Stanford University.

**Results:**

The positive blood isolate was the most prevalent infection site (32.37%) followed by sputum and urine isolates. Gram-negative microorganisms (74.41%) were the most common pathogens, with *Klebsiella pneumoniae* as the most frequently identified one with an incidence of 33.51% followed by *Escherichia coli* with 19.3% incidence. Antibiotic sensitivity showed that colistin is the most effective antibiotic with 96.2%, 94.7%, and 89.9% sensitivity for Klebsiella, *E. coli*, and Acinetobacter, respectively, while carbepenems sensitivity was extremely low, showing 19.5% and 19% imipenem and meropenem sensitivity for Klebsiella, 48% imipenem and 52.7% meropenem sensitivity for *E. coli*, 20.1% imipenem and 20.3% meropenem sensitivity for Acinetobacter, and 17.3% imipenem and 15.2% meropenem sensitivity for *Pseudomonas aeruginosa*. Fungal infection in our results represented less than 1%.

**Conclusion:**

Our study provides a local baseline epidemiological data which describes the extent of the ICU infections problem in this tertiary care hospital.

**Trial registration:**

ClinicalTrials.gov (NCT04318613)

## Background

Nosocomial infections are a major public health concern these days and a cause of considerable mortality and morbidity for hospitalized patients. They occur among 7–12% of the hospitalized patients globally, with more than 1.4 million people suffering from the infectious complications acquired in the hospital [1]. This problem is aggravated by inadequate infection control in developing countries due to poor hygiene, resource and structural constraints, deficient surveillance data, and lack of awareness regarding nosocomial infections [2].

The highest prevalence of hospital-acquired infections (HAIs) is in intensive care units (ICUs), and it is associated with considerable negative impact on the patients’ outcome with a marked increase in the treatment costs. Therefore, early appropriate antibiotic therapy is a fundamental part of the treatment of these patients, and it can be lifesaving. However, bacteria are becoming more resistant with alarming rates of antibiotic resistance worldwide [3–5].

Antibiotic resistance is part of a broader threat called antimicrobial resistance (AMR) that includes resistance to medicines used to treat all types of infections, including those caused by bacteria, parasites, and fungi [6]. ICUs are considered the epicenter of AMR development due to the severity of critical illness; patients are at high risk of becoming infected through the use of invasive devices (e.g., endotracheal tubes and vascular and urinary catheters) and the extensive antibiotic use with variable infection control practices. Consequently, management of infections in the ICU is a growing challenge, and ICU physicians should have regularly updated antibiograms in order to guide appropriate decisions about the choice of empirical antibiotics when waiting for culture results [6–8].

Antibiograms are reports that summarize the information of bacterial antibiotic susceptibility rates within a single facility over the duration of one calendar year. It is used in tracking bacterial resistance and guiding empirical antibiotics prescription within the facility [9].

With the high burden of AMR and the ample variety between ICUs in the prevalence of microorganisms and their antibiotic susceptibility, it is crucial that the selection of empirical antibiotic therapy should be guided by an ICU-specific antibiogram. Also, the emerging trends in bacterial resistance at the local level should be monitored regularly [10].

*The aim of this study* was to find out the prevalence and types of pathogens and to determine their antibiotic susceptibility and resistance in different ICUs of an Egyptian tertiary care hospital (Zagazig University Hospitals).

## Methods

### Study design and setting

This retrospective analytical record-based cross-sectional study was carried out over a 1-year period from the first of January to the last of December 2019 with a total of 45,221 clinical isolates obtained from various clinical samples from different ICUs in Zagazig University Hospitals.

Zagazig University Hospitals are tertiary care teaching hospitals that serve East Delta, Sinai, and Suez Canal governorates. Its intensive care units include Emergency (20 beds), Surgical (32 beds), Medical (50 beds), Pulmonary (15 beds), Coronary (16 beds), Neonatal (40 beds), Pediatric (15 beds), and Cardiothoracic (9 beds) ICUs. All included patients in these ICUs were suffering from signs and symptoms of infection during the study period.

Patients were diagnosed based on clinical presentation, and they were subjected to full clinical history taking with focus on associated risk factors such as hospital stay duration, underlying medical conditions, and invasive medical procedures. The diagnostic criteria and all investigations were performed following relevant local guidelines, protocols, and regulations, and all the data were extracted from the laboratory information system (LIS).

Diagnostic first isolate culture/patient with verified final results and routinely tested antimicrobial agents were included in this study, while we excluded surveillance cultures and screening isolates, duplicate bacterial isolates, and those with reported intermediate sensitivity.

Culture specimens were collected from blood, urine, endotracheal secretions, sputum, bronchoalveolar lavage (BAL), central venous catheter tips, pus, wounds/surgical site swabs, cerebrospinal fluid (CSF), and peritoneal and pleural fluids.

Blood cultures were done using automated Bact/ALERT3D microbial detection system (BioMerieux Inc, Durham, USA) and incubated for 7 to 10 days. Positive blood culture bottles and other isolated samples were initially grown on blood agar and MacConkey and Sabouroud agars for 24 to 48 h at 37 °C.

Colonies appeared and recognized by Gram staining were identified using matrix-assisted laser desorption ionization-time of flight mass spectrophy (MALDI-TOF MS) (BioMerieux, Marcy l'Etoile, France) according to manufacturer instructions. Then colonies were picked and smeared on the wells of disposable target slides; 1 μL of formic acid was added for Candida colonies and air dried then 1 μL VITEC MS CHCA matrix solution (cyano-4-hydroxycinnamic acid) was added to the sample and left to dry at room temperature for 1–2 min then the target slide was loaded into VITEC MS; the mass spectra acquired for each sample were compared with the known mass spectra in the database given a confident score.

Susceptibility of Antimicrobial agents was tested on Vitek 2 Susceptibility cards provided by the manufacturer (Biomerieux, Marcy l’etoile, France); (GN 71, GN 204) for Gram-Negative bacilli, (GN 222) for Gram-Negative resistant strains, (GP 67) for Gram-Positive bacilli, and for yeast card no (AST/ Y S07).

Results were interpreted according to the Clinical and Laboratory Standards Institute (CLSI) 2019 criteria [11], and it was interpreted as sensitive (S), intermediate (I), and resistant (R). The antibiogram construction was done according to CLSI instructions and a Web-based antibiogram at Stanford University [12].

### Ethical approval and trial registration

Our study was approved by the research ethical committee of Faculty of Medicine, Zagazig University with the reference number ZU-IRB#: 5944-5-3-2020, and it was registered with ClinicalTrials.gov (NCT04318613).

### Statistical analysis

Statistical analyses were performed using Excel version 2010 (Microsoft Corporation, USA). Categorical data were presented as percent susceptibility for each antimicrobial agent tested.

## Results

A total of 45,221 clinical isolates were obtained from various clinical samples from different ICUs during the study period. Positive isolates from blood indicating bacteremia was the most prevalent infection site in our ICUs (32.37%) followed by sputum and urine isolates with 28.98% and 16.32% prevalence, respectively. The positive blood samples were the commonest in all ICUs except in emergency, pulmonary, and pediatric ICUs, where positive sputum isolates of ventilated patients were the commonest site (Table 1).

**Table 1:** Prevalence of positive samples in different ICUs

Variables	Emergency ICU (*n* = 11,179)	SICU (*n* = 9528)	MICU (*n* = 20,261)	Pulmonary ICU (*n* = 419)	CCU (*n* = 599)	NICU (*n* = 1582)	PICU (*n* = 1545)	Cardiothoracic ICU (*n* = 108)	Total (*n* = 45,221)
Blood Number (%)	1882 (4.16%)	2672 (5.90%)	8079 (17.86%)	40 (0.08%)	328 (0.72%)	1498 (3.31%)	77 (0.17%)	61(0.13%)	14,637 (32.37%)^a^
Sputum Number (%)	3386 (7.48%)^b^	2826 (6.24%)	5532 (12.23%)	202 (0.44%)^b^	222 (0.49%)	49 (0.10%)	865 (1.91%)^b^	24 (0.05%)	13,106 (28.98%)
Urine Number (%)	646 (1.42%)	1596 (3.52%)	4554 (10.07%)	145 (0.32%)	34 (0.07)	18 (0.03%)	386 (0.85%)	0 (0%)	7379(16.32%)
Wound swab Number (%)	807 (1.78%)	257 (0.56%)	161 (0.35%)	0 (0%)	15 (0.03%)	0 (0%)	0 (0%)	0 (0%)	1240 (2.74%)
Pus Number (%)	2540 (5.61%)	1308 (2.89%)	1352 (2.89%)	0 (0%)	0 (0%)	17 (0.03%)	109 (0.24%)	23 (0.05%)	5349 (11.83%)
Pleural fluid Number (%)	60 (0.13%)	0 (0%)	63 (0.13%)	32 (0.07%)	0 (0%)	0 (0%)	0 (0%)	0 (0%)	155 (0.34%)
Peritoneal fluid Number (%)	100 (0.22%)	48 (0.10%)	231 (0.51%)	0 (0%)	0 (0%)	0 (0%)	33 (0.07%)	0 (0%)	412 (0.91%)
CSF Number (%)	142 (0.31%)	48 (0.10%)	0 (0%)	0 (0%)	0 (0%)	0 (0%)	0 (0%)	0 (0%)	190 (0.42%)
BAL Number (%)	488 (1.07%)	16 (0.03%)	0 (0%)	0 (0%)	0 (0%)	0 (0%)	0 (0%)	0 (0%)	504 (1.11%)
CVC tip Number (%)	1128 (2.49%)	757 (1.67%	206 (0.45%)	0 (0%)	0 (0%)	0 (0%)	75 (0.16%)	0 (0%)	2166 (4.79%)
Vaginal swab Number (%)	0 (0%)	0 (0%)	33 (0.07%)	0 (0%)	0 (0%)	0 (0%)	0 (0%)	0 (0%)	33 (0.07%)
Stool Number (%)	0 (0%)	0 (0%)	50 (0.11%)	0 (0%)	0 (0%)	0 (0%)	0 (0%)	0 (0%)	50 (0.11%)

*ICU* intensive care unit, *SICU* surgical intensive care unit, *MICU* medical intensive care unit, *CCU* coronary care unit, *NICU* neonatal intensive care unit, *PICU* pediatric intensive care unit, *CSF* cerebrospinal fluid, *BAL* bronchoalveolar lavage, *CVC* central venous catheter

*n* total number of clinical isolates in each ICU

Data were expressed as number and percentage. Percentages are out of total isolates.

^a^Bacteremia was the most prevalent infection.

^b^Positive sputum isolates was the commonest infection site in the emergency, pulmonary, and pediatric intensive care units

**Fig. 1:**
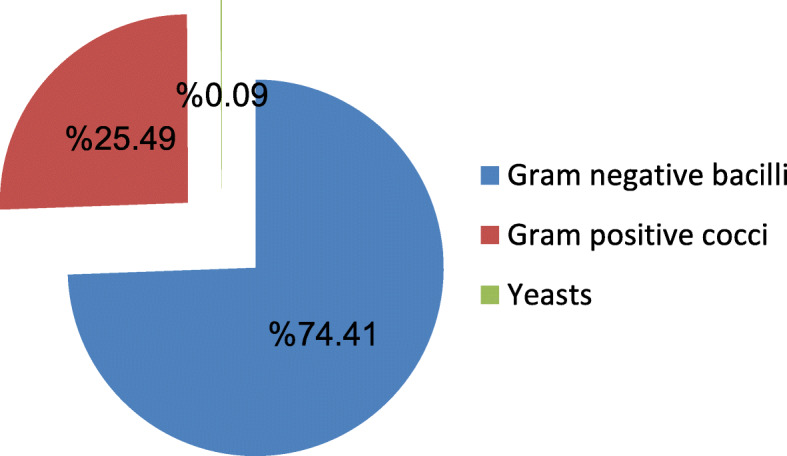
Incidence of microorganisms in all ICUs

The most common pathogens isolated were Gram-negative microorganisms (74.41%) (Fig. 1). Among the array of Gram-negative organisms, *Klebsiella pneumoniae* (*K. pneumoniae*) was the most frequently identified as one with an incidence of 33.51% followed by *Escherichia coli* (*E. coli*) with 19.3% incidence. *K. pneumoniae* was the most prevalent organism in all ICUs except in the CCU, where the Gram-positive cocci were the commonest organisms (Table 2).

**Table 2:** Incidence of isolated microorganisms in different ICUs

Variables	Emergency ICU	SICU	MICU	Pulmonary ICU	CCU	NICU	PICU	Cardiothoracic ICU	Total
	(***n*** = 11,179)	(***n*** = 9528)	(***n*** = 20,261)	(***n*** = 419)	(***n*** = 599)	(***n*** = 1582)	(***n*** = 1545)	(***n*** = 108)	(***n*** = 45,221)
**Microorganisms**									
**Gram-negative Number (%)**	9254 (20.46%)	7952 (17.58%)	13506 (29.86%)	313 (0.69%)	264 (0.58%)	826 (1.83%)	1457 (3.22%)	80 (0.18%)	33,652 (74.41%)
***Acinetobacter baumanii***	1947 (4.30%)	897 (1.98%)	1629 (3.6%)	12 (0.02%)	57 (0.12%)	13 (0.02%)	168 (0.37%)	0 (0%)	4723 (10.44%)
***Burkholderia cepacia***	25 (0.05)	16 (0.03%)	113 (0.24%)	16 (0.03%)	0 (0%)	0 (0%)	0 (0%)	0 (0%)	170 (0.38%)
***Citrobacter freundii***	15(0.03)	17 (0.03%)	16 (0.03%)	0 (0%)	0 (0%)	0 (0%)	0 (0%)	0 (0%)	48 (0.11%)
***E. coli***	2161 (4.77)	1293 (0.02%)	4441 (9.82%)	93 (0.2%)	58 (0.12%)	320 (0.7%)	346 (0.76%)	17 (0.03%)	8729 (19.3%)
***Enterobacter cloacae***	161 (0.35%)	91 (0.2%)	110 (0.24%)	0 (0%)	0 (0%)	2 (0.004%)	0 (0%)	0 (0%)	364 (0.8%)
***Klebsiella pneumoniae***	3756 (8.3%)	4071(9.0%)	5760 (12.73%)	160 (0.03)	149 (0.32%)	452 (0.99%)	742 (1.64%)	63 (0.13%)	15,153 (33.51%)^a^
***Morganella morganii***	15 (0.03%)	0 (0%)	0 (0%)	0 (0%)	0 (0%)	0 (0%)	0 (0%)	0 (0%)	15 (0.03%)
***Proteus mirabilis***	269 (0.59%)	466 (1.03%)	269 (0.59%)	0(0%)	0 (0%)	16 (0.03%)	15 (0.03%)	0 (0%)	1035 (2.29%)
***Providencia stuartii***	81 (0.17%)	16 (0.03%)	23 (0.05%)	0 (0%)	0 (0%)	0 (0%)	0 (0%)	0 (0%)	120 (0.27%)
***P. aeruginosa***	793 (1.75%)	1085 (2.39%)	1115 (2.46%)	32 (0.07%)	0 (0%)	8 (0.01)	170 (0.37%)	0 (0%)	3203 (7.08%)
***Serratia marcescens***	31(0.06%)	0 (0%)	30 (0.06%)	0 (0%)	0 (0%)	15 (0.02%)	16 (0.03%)	0 (0%)	92 (0.2%)
**Gram-positive Number (%)**	1912 (4.22%)	1574 (3.48%)	6729 (14.88%)	106 (0.23%)	335 (0.74%)^b^	756 (1.67%)	88 (0.19%)	28 (0.06%)	11,528 (25.49%)
**Enterococci**	144 (0.31%)	91 (0.2%)	651 (1.43%)	26 (0.05%)	26 (0.05%)	13 (0.02%)	13 (0.02%)	12 (0.02%)	976 (2.16%)
***Staph. aureus***	589 (1.3%)	287 (0.63%)	1170 (2.58%)	16 (0.03%)	47 (0.1%)	80 (0.17%)	30 (0.06%)	0 (0%)	2219 (4.91%)
***Staph. hominis***	382 (0.84%)	339 (0.75%)	1860 (4.11%)	16 (0.03%)	139 (0.3%)	295 (0.65%)	16 (0.03%)	0 (0%)	3047 (6.73%)
**Other staph.**	773 (1.7%)	848 (1.87%)	3039 (6.72%)	48 (0.1%)	123 (0.27%)	361 (0.79%)	29 (0.06%)	16 (0.03%)	5237 (11.58%)
**Streptococci**	24 (0.05)	9 (0.01%)	9 (0.01%)	0 (0%)	0 (0%)	7 (0.01%)	0 (0%)	0 (0%)	49 (0.11%)
**Yeasts Number (%)**	13 (0.11%)	2 (0.004%)	26 (0.12%)	0 (0%)	0 (0%)	0 (0%)	0 (0%)	0 (0%)	41 (0.09%)
***Candida albicans***	6 (0.01%)	2 (0.004%)	11 (0.02%)	0 (0%)	0 (0%)	0 (0%)	0 (0%)	0 (0%)	19 (0.04%)
***Candida tropicalis***	7 (0.01%)	0 (0%)	15 (0.03%)	0 (0%)	0 (0%)	0 (0%)	0 (0%)	0 (0%)	22 (0.05%)

*ICU* intensive care unit, *SICU* surgical intensive care unit, *MICU* medical intensive care unit, *CCU* coronary care unit, *NICU* neonatal intensive care unit, *PICU* pediatric intensive care unit, *E. coli Escherichia coli*, *P. aeruginosa Pseudomonas aeruginosa*, *Staph. aureus Staphylococcus aureus*, *Staph. hominis Staphylococcus hominis*

*n* total number of clinical isolates in each ICU

Data were expressed as number and percentage. Percentages are out of total isolates.

Data from isolate totals < 30 may be statistically unreliable.

^a^*Klebsiella pneumoniae* was the most frequently identified Gram-negative microorganism.

^b^Gram-positive cocci was more common in CCU versus Gram-negative organisms

Staphylococcus species was isolated in 10,503 isolates with *Staph. hominis* as the commonest isolated Staph*.* species (29% of Staph. isolates, 6.73% of total isolates, *n* = 3047) (Table 2) and *Staph. haemolyticus* was the 2nd commonest Staph. species (28.12% of Staph. isolates, 6.53% of total isolates, *n* = 2954), while *Staphylococcus aureus* (*Staph. aureus*) was isolated in 21.1% of Staph. isolates (4.91% of all isolates, *n* = 2219) with the methicillin-resistant *Staph. aureus* (MRSA) found in 18.98% of Staph. isolates (4.4% of all isolates, *n* = 1994 isolates), while methicillin-sensitive *Staph. aureus* (MSSA) accounts for only 2.14% of Staph. species (*n* = 225 isolates, 0.49% of all isolates) in our study.

Gram-positive cocci was the commonest organism isolated in blood cultures and *K. pneumoniae* the commonest pathogen isolated from sputum cultures. Also, *E. coli* was the most common isolated organism from urine cultures (Table 3).

**Table 3:** Incidence of isolated microorganisms in different samples

Cultures (***n*** = 45,221)	Gram-negative bacteria (***n*** = 33,652)											Gram-positive bacteria (***n*** = 11,528)				Candida (***n*** = 41)	
	***Acinetobacter baumanii*** (***n*** = 4723)	***Burkholderia cepacia*** (***n*** = 170)	***Citrobacter freundii*** complex (***n*** = 48)	***Enterobacter cloacae*** (***n*** = 364)	***E. coli*** (***N*** = 8729)	***K. pneumoniae*** (***n*** = 15153)	***Proteus mirabilis*** (***n*** = 1035)	***P. aeruginosa*** (***n*** = 3203)	***Morganella morganii*** (***n*** = 15)	***Providencia stuartii*** (***n*** = 120)	***Serratia marcescens*** (***n*** = 92)	Streptococci (***n*** = 49)	Enterococci (***n*** = 976)	***Staph. aureus*** (***n*** = 2219)	Other Staphylococci (***n*** = 8284)	***C. albicans*** (***n*** = 19)	***C. tropicalis*** (***n*** = 22)
**Blood culture**	866	42	0	99	1602	2696	168	456	0	0	60	13	513	931	7205*	6	10
**CVC tip**	343	0	9	58	220	850	93	97	0	47	0	0	13	177	236	0	0
**Sputum**	1949	16	0	37	2319	6421	170	1044	0	17	16	0	13	745	429	0	1
**BAL**	195	0	0	0	76	184	0	17	0	0	0	0	0	16	16	0	0
**CSF**	28	0	0	0	17	114	0	15	0	0	0	0	0	0	16	0	0
**Pus**	737	0	0	31	1107	1962	365	634	15	33	16	12	13	209	138	0	0
**Urine**	428	112	24	77	2909♦	2308	178	615	0	23	0	21	405	64	213	13	11
**Wound swab**	135	0	15	62	211	437	49	239	0	0	0	0	0	45	15	0	0
**Pleural fluid**	42	0	0	0	33	16	0	48	0	0	0	0	0	16	0	0	0
**Peritoneal fluid**	0	0	0	0	201	132	16	22	0	0	0	9	0	16	16	0	0
**Vaginal swab**	0	0	0	0	0	17	0	16	0	0	0	0	0	0	0	0	0
**Stool**	0	0	0	0	34	16	0	0	0	0	0	0	0	0	0	0	0

*CVC* central venous catheter, *BAL* bronchoalveolar lavage, *CSF* cerebrospinal fluid

Data were expressed as number

^*^Gram-positive cocci were the commonest organism isolated in blood cultures

¶ *K. pneumoniae* the commonest organism isolated in sputum cultures

♦ *E. coli* the commonest organism isolated from urine cultures

**Fig. 2:**
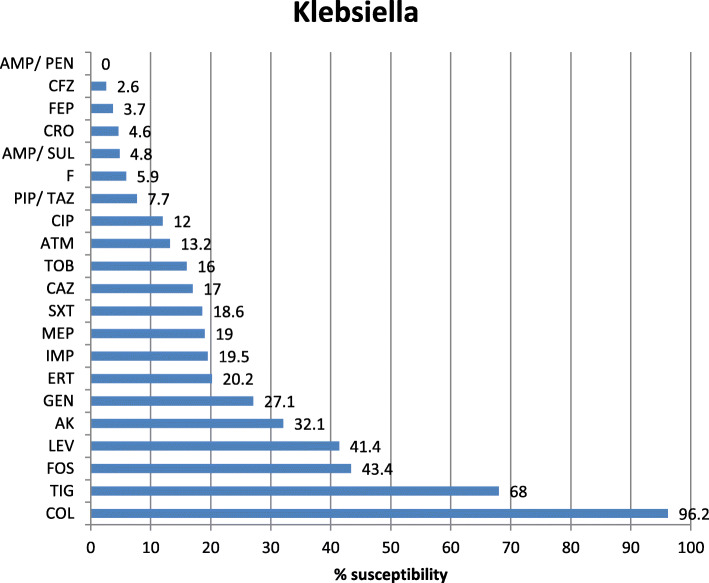
Antibiotic susceptibility of the *Klebsiella pneumoniae*. *Klebsiella Klebsiella pneumoniae*, *AMP/PEN* ampicillin/penicillin, *CFZ* cefazolin, *FEP* cefepime, *CRO* ceftriaxone, *AMP/SUL* ampicillin/sulbactam, *F* nitrofurantoin, *PIP/TAZ* pipracillin/tazobactam, *CIP* ciprofloxacin, *ATM* aztreonam, *TOB* tobramycin, *CAZ* ceftazidime, *SXT* sulfa-trimethoprim, *MEP* meropenem, *IMP* imipenem, *ERT* ertapenem, *GEN* gentamycin, *AK* amikacin, *LEV* levofloxacin, *FOS* fosfomycin, *TIG* tigecylcine, *COL* colistin

**Fig. 3:**
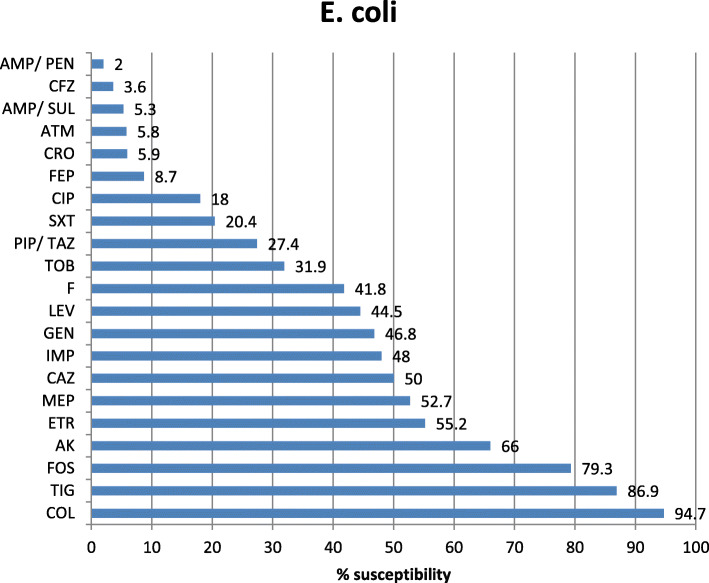
Antibiotic susceptibility of *Escherichia coli*. *E. coli Escherichia coli*, *AMP/PEN* ampicillin/penicillin, *CFZ* cefazolin, *FEP* cefepime, *CRO* ceftriaxone, *AMP/SUL* ampicillin/sulbactam, *F* nitrofurantoin, *PIP/TAZ* pipracillin/tazobactam, *CIP* ciprofloxacin, *ATM* aztreonam, *TOB* tobramycin, *CAZ* ceftazidime, *SXT* sulfa-trimethoprim, *MEP* meropenem, *IMP* imipenem, *ERT* ertapenem, *GEN* gentamycin, *AK* amikacin, *LEV* levofloxacin, *FOS* fosfomycin, *TIG* tigecylcine, *COL* colistin

**Fig. 4:**
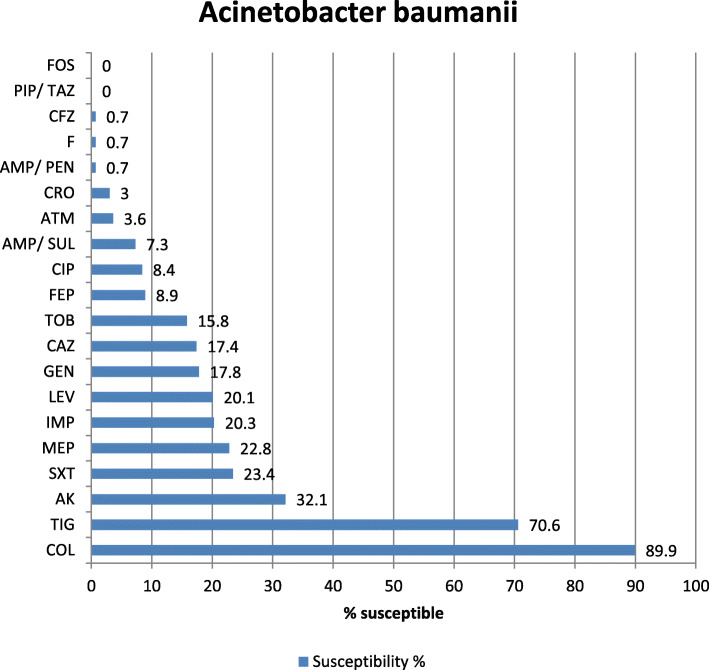
Antibiotic susceptibility of *Acinetobacter baumanii*. *AMP/PEN* ampicillin/penicillin, *CFZ* cefazolin, *FEP* cefepime, *CRO* ceftriaxone, *AMP/SUL* ampicillin/sulbactam, *F* nitrofurantoin, *PIP/TAZ* pipracillin/tazobactam, *CIP* ciprofloxacin, *ATM* aztreonam, *TOB* tobramycin, *CAZ* ceftazidime, *SXT* sulfa-trimethoprim, *MEP* meropenem, *IMP* imipenem, *ERT* ertapenem, *GEN* gentamycin, *AK* amikacin, *LEV* levofloxacin, *FOS* fosfomycin, *TIG* tigecylcine, *COL* colistin

**Fig. 5:**
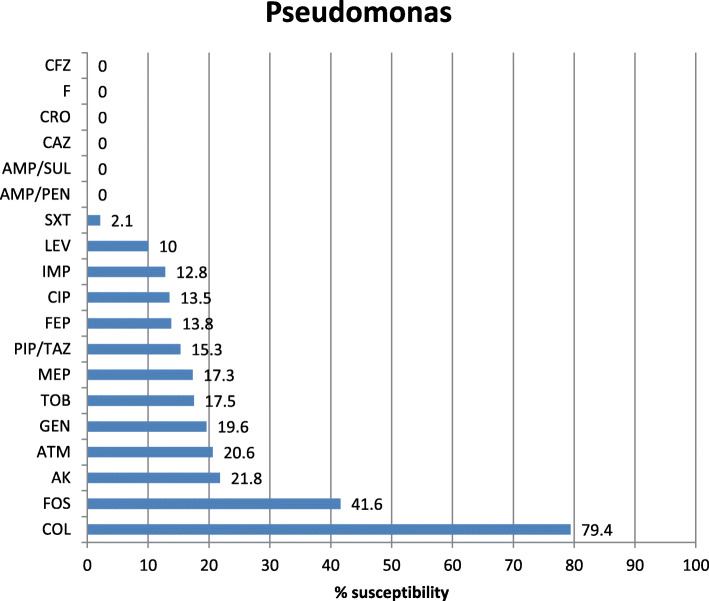
Antibiotic susceptibility of *Pseudomonas aeruginosa*. *Pseudomonas Pseudomonas aeruginosa*. *AMP/PEN* ampicillin/penicillin, *AMP/SUL* ampicillin/sulbactam, *PIP/TAZ* pipracillin/tazobactam, *CAZ* ceftazidime, *CRO* ceftriaxone, *FEP* cefepime, *ATM* aztreonam, *IMP* imipenem, *MEP* meropenem, *ERT* ertapenem, *GEN* gentamycin, *TOB* tobramycin, *AK* amikacin, *CIP* ciprofloxacin, *LEV* levofloxacin, *SXT* sulfa-trimethoprim, *COL* colistin, *TIG* tigecylcine, *FOS* fosfomycin, *F* nitrofurantoin, *CFZ* cefazolin

**Fig. 6:**
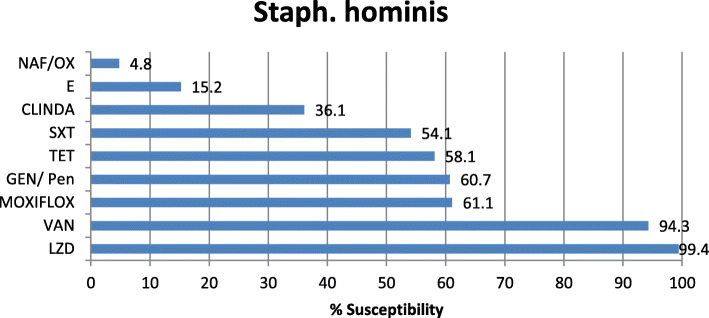
Antibiotic susceptibility of Gram-positive *Staph. hominis*. *NAF/OX* nafcillin/oxacillin, *E* erythromycin, CLINDA clindamycin, *SXT* sulfa-trimethoprim, *TET* tetracycline, *GEN/pen* gentamycin/penicillin, *MOXIFLOX* moxifloxacillin, *VAN* vancomycin, *LZD* linezolid

**Fig. 7:**
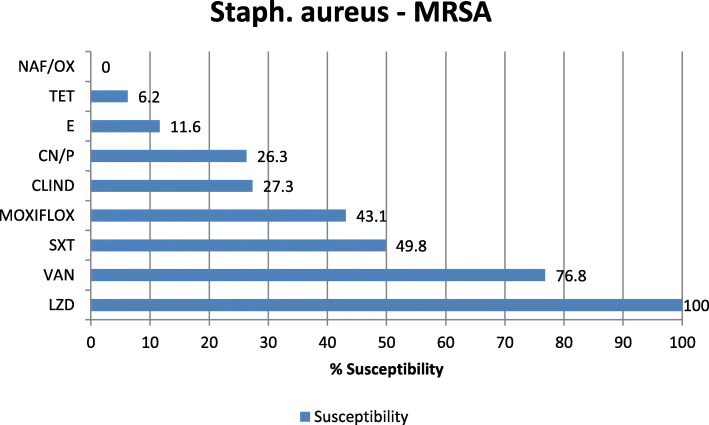
Antibiotic susceptibility of methicillin-resistant *Staph. aureus*. *MRSA* methicillin-resistant *Staph. aureus*, *NAF/OX* nafcillin/oxacillin, *E* erythromycin, *CLINDA* clindamycin, *SXT* sulfa-trimethoprim, *TET* tetracycline, *GEN/pen* gentamycin/penicillin, *MOXIFLOX* moxifloxacillin, *VAN* vancomycin, *LZD* linezolid

Antibiotic sensitivity of the most commonly isolated Gram-negative pathogens in our study was highly variable showing that colistin is the most effective antibiotic with 96.2, 94.7, and 89.9% sensitivity for *K. pneumoniae*, *E. coli*, and Acinetobacter, respectively. The tigecycline sensitivity was 86.9% for *E. coli*, 70.6% for Acinetobacter, and 68% for *K. pneumoniae* while carbepenem sensitivity for these organisms was extremely low showing 19.5% and 19% imipenem and meropenem sensitivity of *K. pneumoniae*, 48% imipenem and 52.7% meropenem sensitivity of *E. coli*, 20.1% imipenem and 20.3% meropenem sensitivity of Acinetobacter, and 17.3% imipenem and 15.2% meropenem sensitivity of *P. aeruginosa* (Figs. 2, 3, 4, and 5). The vancomycin sensitivity of Gram-positive *Staph. hominis* was 94.3%, and it was 76.8% for MRSA (Figs. 6 and 7).

**Fig. 8:**
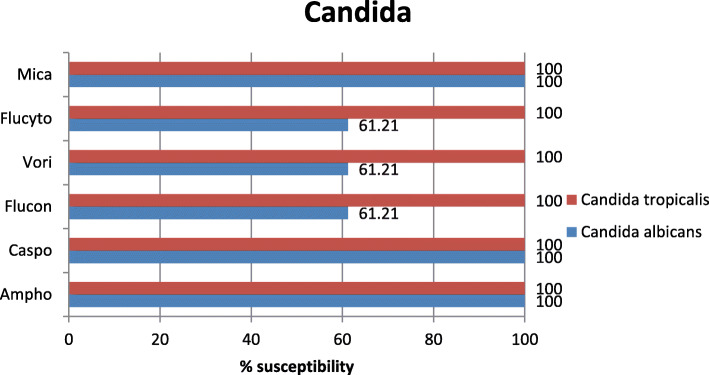
Antifungal susceptibility of *Candida* species. *Ampho* amphotericin B, *Caspo* caspofungin, *Flucon* fluconazole, *Vori* voriconazole, *Flucyto* flucytosine, *Mica* micafungin

Fungal infection in our results represented less than 1% mostly *C. tropicalis* 0.05% vs 0.04% *C. albicans* with the antifungal sensitivity of *C. tropicalis* is around 100% for all antifungal classes, while the azole sensitivity of *C. albicans* was 61.2% for both fluconazole and vorioconazole (Fig. 8).

## Discussion

The use of antibiograms to help select empirical antibiotic therapy for suspected infection with likely or known pathogens is a well-established practice. This is the first study to describe the analysis of antibiogram results and provide the epidemiological information of microorganisms and antibiotics for the Zagazig University multidisciplinary ICUs. Our study dealt with the analysis of culture-sensitivity reports of only ICU patients which was taken either on patients’ admission with community- or hospital-acquired infection (Emergency Department (ED) admission or ward transfer), or after ICU-acquired infections.

Our results reported that bacteremia was the most prevalent infection (32.37%) in our university hospital ICUs which is against the widely recognized that catheter-associated urinary tract infection (CAUTI) is the most common hospital-acquired infection (HAI) in the world, accounting for 40% of all HAIs [13]. This could be explained by the inclusion of only ICU patients in our study, not all hospitalized patients. Also, the well-known high utilization of central venous lines in critically ill patients could increase the risk of bacteremia; moreover, some studies found higher device-associated infection (DAI) rates in teaching hospitals when compared with nonteaching hospitals [14, 15]. Also, contrary to our results, Shebl et al., in their study, found that out of 554 bacterial isolates, urine specimens showed the highest incidence of total isolates (41.5%, *n* = 230) followed by blood (23.1%, *n* = 128), while sputum specimens exhibited the least frequency (17%, *n* = 94) [16]. Also, Klevens et al. reported that urinary tract infection (UTI) accounts for more than 30% of infections in acute care hospitals [17], while respiratory tract infection (RTI) accounted for 64.75% of total nosocomial infections (NIs) in Shao et al.’s study followed by UTI which accounted for 9.4% and bloodstream infection (BSI) for 7.96% [18]. This should raise our need to review our clinical protocols for localization of infection and rationale of blood cultures together with asking about the methods of proper blood sampling to exclude the contamination in our ICUs.

In the current study, we found that in the emergency, pulmonary and pediatric intensive care units, positive sputum isolates were the commonest. Local surveillance of these units based on clinical, radiological, plus laboratory findings showed that ventilator-associated pneumonia (VAP) was the commonest versus those from the blood bacteremia (7.48%, *n* = 3386 vs 4.16%, *n* = 1882; 0.44%, *n* = 202 vs 0.08%, *n* = 40; and 1.91%, *n* = 865 vs 0.17%, *n* = 77 for emergency, pulmonary, and pediatric ICUs, respectively) which could be attributed to the different characters of the patients admitted to these units as most of them are in severe critical conditions with respiratory failure, multiple trauma, multiple organ failure, septic shock, or after cardiopulmonary resuscitation; mostly, they are in a coma-like state, with a decreased cough reflex; drainage is impeded, or endotracheal intubation is used along with a ventilator.

Gram-negative bacterial infections have been recently reported to be significantly increased worldwide. Our results confirmed that the most common pathogens isolated were Gram-negative bacteria (74.41%), which may be due to their wide prevalence in the hospital environment. Additionally; their frequent resistance to antibiotics may play a role in their persistence and spread. Among the array of Gram-negative organisms, *K. pneumoniae* was the most frequently identified one, and this is the same in all units except for the coronary care unit (CCU).

This is in agreement with the US National Healthcare Safety Network recent data indicating that Gram-negative bacteria are accountable for more than 30% of hospital-acquired infections [19]. Similar to our study, Klebsiella was the most predominant organism in Rajan and Rao’s study [20], while *Pseudomonas aeruginosa* was reported to be the predominant ICU isolates in Al-Ahmadey et al.’s study [21]. Qadeer et al., in their study, reported that Acinetobacter and *E. coli* were the predominant isolates [22]. Similarly, Al-Jawady et al. [23] and Morfin-Ortero et al. [24] found that *E. coli* was the most common Gram-negative bacillus. As well as in Shebl et al.’s study, Gram-negative organisms were the most common isolates (68.4%) with the predominance of *E. coli* (30.7%) followed by Klebsiella species (20.9%), while *Staph. aureus* accounted for 21.1% in their study [16].

Our study found that *K. pneumoniae* was the most common isolate from respiratory tract, and *E. coli* was most frequently isolated from urine which is in-line with results of many studies [20, 25–27]. However, Pradhan et al. in their study showed that Acinetobacter species were the most frequent microorganisms in the respiratory tract [28] as well as in Kanj et al.’s study which reported Acinetobacter species as the commonest VAP isolates, and *E. coli* was the most common isolate from both central line-associated bloodstream infection (CLA-BSI) and CAUTI [29].

Institution-wide antibiograms may conceal important differences in susceptibility data across units within the institution. These differences may be significant, not only for selecting the most effective empirical antimicrobial therapy for a patient in that unit but also for monitoring the emerging patterns of antimicrobial resistance specific to certain units within the institution [30]. In the present study, antibiograms were segregated on the basis of different units where patients were admitted.

In our Pulmonary ICU, the most common organism was *K. pneumoniae*. This finding is not in agreement with reports from the USA which suggest that *P. aeruginosa* is the most frequent bacterium isolated from the respiratory tract (31.6%) [31], and also, the Egyptian study by Elkolaly et al. in their results found that the isolated organisms were Pseudomonas (37.5%), Klebsiella (25%), Staphylococcus (20.8%), and methicillin-resistant *Staphylococcus aureus* (4.2%) [32].

The antibiogram of our Surgical ICU showed Gram-negative bacteremia is the predominant infection with the most prevalent organism is *K. pneumoniae*. However, the findings from a study in an adult surgical intensive care unit in the Republic of South Africa showed the high rates of HAI, especially for lower respiratory tract infection (LRTI) (81.8/1000 IP-Days), surgical site infection (SSI) (31.7/1000 IP-Days), and blood stream infection (BSI) (26.4/1000 IP-Days). *Acinetobacter baumannii* was the most common organism representing 31% of all infections [33].

The current study found that in the CCU, Gram-positive cocci were the most commonly identified organisms (0.74%, *n* = 335 Gram-positive vs 0.58%, *n* = 264 Gram-negative organisms). The frequency of coronary catheterization, central device insertion, and infective endocarditis may explain the prevalence of Gram-positive bacteria in the CCU.

Supporting our results in the NICU, Almohammady et al., in their study, identified Gram-negative organisms mainly Klebsiella as the most prevalent organisms among neonatal sepsis cases and Acinetobacter as the second most common isolate; however, in our study, *E. coli* was the second most common isolate [34]. We also observed that the prevalence of Gram-positive organisms in NICU was high (1.67% of total isolates, 48% of NICU isolates, *n* = 756) that could be attributed to the high frequency of prematurity and very low birth weight in the neonatal population which is the most important host risk factors for coagulase-negative staphylococci (CoNS) infection. The greater number of skin breaks for catheter insertion was also considered as a significant predictor of CoNS sepsis as well as total parenteral nutrition (TPN) use with or without a central venous catheter has been demonstrated to be a risk factor [35].

Antibiotics are one of the main pillars of modern medicine and play a vital role both as the prophylaxis and management of infectious diseases. Successful treatment of patients with bacterial infection relies on the identification of bacterial pathogens and on the selection of an antibiotic effective against that particular organism [36]. Unreasonable use of antimicrobials is the biggest contributing factor to the growing threat of resistance especially in low-income countries [37]. It is worth mentioning that antimicrobial therapy should take into account the data regarding the local prevalence of causative pathogens and their antimicrobial resistance profile rather than the universal guidelines.

Klebsiella, the most common microorganism in our study (33.51%), showed high carbapenem resistance (81% meropenem and 80.5% imipenem), while in Qadeer et al.’s study, less resistance was observed (56% meropenem and 55% imipenem) [22], whereas Sheth et al. showed 100% sensitivity to carbapenems [38] and Rajan et al. documented 28.13% carbapenem resistance [20]. In the present study, a high pattern of resistance was seen with third-generation cephalosporins (95.4% ceftriaxone) and 96.3% for cefepime (4th generation) and aminoglycosides (72.9% gentamicin, 67.9% amikacin). Also, in Qadeer et al.’s study, a high pattern of resistance was seen with third-generation cephalosporins (94% ceftazidime, 82% ceftriaxone, and 70% cefoperazone/sulbactam) and aminoglycosides (61% gentamicin, 48% amikacin) [22]. Gunjal et al. has reported 60% resistance to amikacin and 80% resistance to gentamicin [39]. The most effective drug was colistin, which showed 3.8% resistance in our study followed by tigecycline with resistance 32%. Whereas, tigecycline was found to be the effective antibiotic against multidrug-resistant Klebsiella in Qadeer et al.’s study [22].

Our study showed 13.1% tigecycline resistance to *E. coli* (second common organism 19.3%) and also showed high resistance to third-generation cephalosporins (94.1% ceftriaxone) and 91.3 % for cefepime (4th generation). Qadeer et al.’s study showed 33% tigecycline resistance to *E. coli*, and showed high resistance to third-generation cephalosporins (93% ceftazidime and 90% ceftriaxone) [22]; similarly, more than 90% *E. coli* were found to be resistant to third-generation cephalosporin by Al mohammady et al. [34]. Carbapenem resistance was 52% for imipenem and 47.3% for meropenem. Carbapenem resistance was as low as 10% in Qadeer et al. [22]. Almost similar results reported by Bayram et al. [40] showed 13.1% *E. coli* resistance to imipenem. Gunjal et al. [40] reported that 28.10% of *E. coli* isolates were resistant to amikacin and 48.20% resistance to gentamicin, whereas resistance to amikacin and gentamicin were 34% and 53.2%, respectively, in our study. Colistin showed only resistance by 5.3 % to *E. coli* strains in the present study.

Our study shows a very high prevalence of carbapenem resistance among Acinetobacter (third prevalent organism 10.44%), 79.9% for imipenem and 79.7% for meropenem. Qadeer et al.’s study showed 100% resistance to carbapenems [22]. Another study conducted by Khan has reported 79% resistance to imipenem [41], while Rajan et al. showed 52% carbapenem resistance among Acinetobacter [20]. In our study, Acinetobacter was highly resistant to third-generation cephalosporins (97% ceftriaxone), aminoglycosides (82.2% gentamicin and 67.9% amikacin), and quinolones (91.6% ciprofloxacin and 79.9 levofloxacin). In Qadeer et al.’s study, Acinetobacter was also highly resistant to third-generation cephalosporins (100% ceftazidime), aminoglycosides (97% gentamicin and 95% amikacin), and fluoroquinolones (100% ciprofloxacin and moxifloxacin) [22]. The most effective drug was colistin which showed 10.1% resistance followed by tigecycline (29.4%). Also, in Qadeer et al.’s study, the most effective drug was colistin which showed 3% resistance. Similar results of colistin effectiveness against Acinetobacter were seen in the study by Rajan et al. [20], while work published by Hasan et al. [42] showed that tigecycline was the most effective antibiotic against Acinetobacter. Acinetobacter was the second most common Gram-negative isolate showed only 25% sensitivity to levofloxacin and 100% to polymyxin in Almohammadi-Mehr et al.’s study [31].

In our study, Pseudomonas (4th prevalent one 7.08%) showed significant resistance to carbapenems (82.7% imipenem/ 84.7% meropenem). In Qadeer et al.’s study [22], Pseudomonas showed less resistance to carbapenems (59% imipenem/meropenem), whereas a study published by Rakhee et al. [43] showed 20.8% resistance to imipenem and the study published by Rajan et al. [20] showed 12.9% carbapenem resistance to Pseudomonas. Pseudomonas also showed high resistance to third-generation cephalosporins (100% ceftriaxone) and 86.2% to cefepime (4th generation), while aminoglycosides showed (80.4% gentamicin and 78.2% amikacin). In Qadeer et al.’s study [22], Pseudomonas aeruginosa resistance to third- generation cephalosporins was (53% cefoperazone/sulbactam and 39% to ceftazidime) and for aminoglycosides was (48% gentamicin and 41% amikacin). Radji et al. showed 60.9% resistance to ceftriaxone and found that amikacin was the most effective antibiotic against Pseudomonas with 15.6% resistance [44]. We found colistin to be the most effective antibiotic against Pseudomonas with 20.6% resistance.

The most common Gram-positive organism was *Staph. hominis* (coagulase-negative staphylococci): 26.43% of all Gram positive, 29.01% of Staph. species and the fifth one as one of the total organisms (6.73%) where sensitivity to vancomycin and linezolid was 94.3% and 99.4%, respectively. This was followed by *Staph. aureus* (coagulase-positive staphylococci): 4.9% of total organism and 19.24% in between Gram-positive cocci, MRSA was 89.86% of them where sensitivity to vancomycin and linezolid were 76.8 and 100%, respectively. The resistance of MRSA to vancomycin may be because of its prolonged and frequent abuse in empirical use.

Savanur et al. [45], in their study, dedicated that among the Gram-positive organisms; coagulase negative staphylococcus (CoNS) (15.6%) was most commonly isolated followed by Streptococcus (2.32%), while Chidambaram et al. [46] reported that among the Gram-positive isolates, Enterococcus (4.79%) was the common isolate obtained followed by *Staphylococcus aureus* (3.72%).

Fungal growth represented less than 1% in our study. Conversely, Savanur et al. found that fungal growth was seen in 15.11% [45]. This difference may be due to the false-negative records in our hospital which could be because of two main factors: the lack of orientation and the deficient knowledge about the need of fungal investigations in some ICUs; only emergency, surgical, and medical ICUs were investigated after consideration. *Candida albicans* was seen in 22 samples, where *Candida tropicalis* was seen in 19 samples only with a total of 41 with 100% sensitivity to amphotericin B, caspofungin, and micafungin.

The high-resistance pattern reported in our study could be explained by the prior antibiotic usage, prior severe Gram-negative infections, inappropriate course of antibiotics, and patients’ coming with severe sepsis and septic shock as our hospital is a referral tertiary care hospital which increases the possibility of emergence of multidrug-resistance organisms, and this high incidence of resistance should be alarming and highlight the necessity of routine monitoring of the local prevalence of resistance that could help in selecting the best antimicrobial treatment and guide the empirical therapy.

Seeing that we are facing a high, serious, and threatening incidence of antimicrobial resistance with limited choices in empirical antibiotics, a comprehensive program is highly needed to combat this enemy which should be a national priority. This program comprises the implementation of infection control policies, care bundles, antimicrobial stewardship program (ASP), quality, and education. By using the available antibiogram data, the only solutions for our ICUs will be the combination of antibiotics and providing recently available new antibiotic generations in our institution until the successful implementation of ASP, not only in our university hospital ICUs but also all over all Egyptian hospitals.

Our study was limited by the absence of clinical data to distinguish between hospital-acquired and community-acquired infections and the data required to differentiate between true infection and colonization. Another limitation is our dependence on the first-isolate approach in order to reduce the bias that may be present in an all-isolate approach. Although the first-isolate approach is recommended by CLSI, it might underestimate the resistance rate of nosocomial infections.

## Conclusion

In conclusion, our study documented the problem of high rates of ICU infections in Zagazig University Hospitals and it provides a local baseline epidemiological data which describes the extent of the ICU infections problem in this tertiary care hospital that can be used to monitor trends through building a cumulative antibiograms and assess the success of preventive strategies in the future.

This local prevalence study will aid in establishing an effective antimicrobial stewardship to preserve the potentials of the current antimicrobial agents. For example; high resistance of gram negative to carbapenem emphasize the implementation of carbapenem sparing techniques. In order to adequately implement antimicrobial stewardship as a tool to combat antimicrobial resistance in ICUs nationally, further prospective multicentre epidemiological studies are needed at multidisciplinary ICUs.

## Data Availability

The datasets used and/or analyzed during the current study are available from the corresponding author on reasonable request.

## Abbreviations

AMR: Antimicrobial resistance
ASP: Antimicrobial stewardship program
BAL: Bronchoalveolar lavage
BSI: Bloodstream infection
CAUTI: Catheter-associated urinary tract infection
CCU: Coronary care unit
CLA-BSI: Central line-associated bloodstream infection
CLSI: Clinical and Laboratory Standards Institute
CSF: Cerebrospinal fluid
CVC: Central venous catheter
DAI: Device-associated infection
ED: Emergency Department
*E. coli*: *Escherichia coli*
HAI: Hospital-acquired infection
ICU: Intensive care unit
LIS: Laboratory information system
MICU: Medical intensive care unit
NICU: Neonatal intensive care unit
PICU: Pediatric intensive care unit
*P. aeruginosa*: *Pseudomonas aeruginosa*
RTI: Respiratory tract infection
SICU: Surgical intensive care unit
*Staph. aureus*: *Staphylococcus aureus*
*Staph. hominis*: *Staphylococcus hominis*
VAP: Ventilator-associated pneumonia
UTI: Urinary tract infection
AMP/PEN: Ampicillin/penicillin
AMP/SUL: Ampicillin sulbactam
PIP/TAZ: Pipracillin tazobactam
CAZ: Ceftazidime
CRO: Ceftriaxone
FEP: Cefepime
ATM: Aztreonam
IMP: Imipenem
MEP: Meropenem
ERT: Ertapenem
GEN: Gentamycin
TOB: Tobramycin
AK: Amikacin
CIP: Ciprofloxacin
LEV: Levofloxacin
SXT: Sulfa-trimethoprim
COL: Colistin
TIG: Tigecycline
FOS: Fosfomycin
F: Nitrofurantoin
CFZ: Cefazolin
GEN/pen: Gentamycin/penicillin
VAN: Vancomycin
E: Erythromycin
CLINDA: Clindamycin
LZD: Linezolid
MOXIFLOX: Moxifloxacillin
NAF/OX: Nafcillin/oxacillin
TET: Tetracycline
Ampho: Amphotericin B
Caspo: Caspofungin
Flucon: Fluconazole
Vori: Voriconazole
Flucyto: Flucytosine
Mica: Micafungin
